# Model-Based Feedforward Control of Part Height in Directed Energy Deposition

**DOI:** 10.3390/ma14020337

**Published:** 2021-01-11

**Authors:** Qian Wang, Jianyi Li, Abdalla R. Nassar, Edward W. Reutzel, Wesley F. Mitchell

**Affiliations:** 1 Department of Mechanical Engineering, The Pennsylvania State University, University Park, PA 16802, USA; gejianyili@gmail.com; 2 Applied Research Laboratory, The Pennsylvania State University, University Park, PA 16802, USA; arn5000@arl.psu.edu (A.R.N.); ewr101@arl.psu.edu (E.W.R.); wfm11@arl.psu.edu (W.F.M.)

**Keywords:** directed energy deposition, additive manufacturing, feedforward control, nonlinear inverse-dynamics control, build height regulation

## Abstract

Control of the geometric accuracy of a metal deposit is critical in the repair and fabrication of complex components through Directed Energy Deposition (DED). This paper developed and experimentally evaluated a model-based feedforward control of laser power with the objective of achieving the targeted part height in DED. Specifically, based on the dynamic model of melt-pool geometry derived from our prior work, a nonlinear inverse-dynamics controller was derived in a hatch-by-hatch, layer-by-layer manner to modulate the laser power such that the melt-pool height was regulated during the simulated build process. Then, the laser power trajectory from the simulated closed-loop control under the nonlinear inverse-dynamics controller was implemented as a feedforward control in an Optomec Laser-Engineered Net Shape (LENS) MR-7 system. This paper considered the deposition of L-shaped structures of Ti-6AL-4V as a case study to illustrate the proposed model-based controller. Experimental validation showed that by applying the proposed model-based feed-forward control for laser power, the resulting build had 24–42% reduction in the average build height error with respect to the target build height compared to applying a constant laser power through the entire build or applying a hatch-dependent laser power strategy, for which the laser power values were obtained from experimental trial and error.

## 1. Introduction

Additive Manufacturing (AM), also called 3D printing, refers to a process that builds three-dimensional parts directly from the complex Computer-Aided Design (CAD) files by depositing material in a layer-by-layer manner. Directed energy deposition (DED) is one type of AM processes, where focused energy, such as an electron beam or laser beam, is used to melt material as filler material is being deposited [1,2,3]. Laser-Engineered Net Shape (LENS) and Laser Metal Deposition-powder (LMD-p) are among the typical DED AM processes.

For fabrication of complex components through DED, model-based real-time control or optimization of process parameters is often required to ensure build quality and geometric accuracy of a build part. Most of the existing real-time controllers for DED were restricted to classical controllers, e.g., on/off bang-bang controller [4], and Proportional–Integral–Derivative (PID) controllers [5,6,7,8,9,10,11], to name a few. The PID controllers were designed either based on pure sensing information without a model [6,7,10] or based on relatively simplistic single-input single-output empirical models, e.g., a first-order transfer function [8]; a first-order transfer function plus a time delay [11]; or a knowledge-based empirical model [5,9]. Advanced control methodologies were also investigated in several studies. A fuzzy logic controller was designed to follow the desired clad height in a cladding process by controlling the laser power [12]. A sliding mode controller, together with a PID controller, was developed in [13] for clad height and nozzle standoff distance, based on an empirical model consisting of linear dynamics and a nonlinear memoryless output, with model parameters identified offline using experimental data. A generalized predictive controller was designed based on a second-order empirical model [14,15]. A multivariable control was applied to simultaneously control of the laser power and scan speed in regulating both the melt-pool geometry and melt-pool average temperature for single-tracks in a numerical study for DED [16]. For laser metal deposition, an Iterative Learning Control (ILC) was developed for height control by varying powder flow rate in [17,18], and for melt-pool temperature control by varying laser power in [19]. Iterative learning control was also applied to height control in a laser metal wire deposition process [20], where ILC was used to reduce height deviations by controlling the wire feed rate, based on an empirical first-order transfer function. Readers are referred to the review paper in [21] and references therein.

In contrast to the aforementioned feedback control that required in situ sensing data [22,23,24], several recent studies focused on model-based feedforward control, mainly for the Laser Powder Bed Fusion (L-PBF) systems [25,26,27,28,29], as the feedforward control did not require real-time in situ sensing, but instead only utilized process models or simulations to derive the trajectories of process parameters before building a part. As a result, compared to feedback control, model-based feedforward controllers potentially have fewer barriers for industrial adoption as they require only to load the precomputed trajectories of process parameters to the AM system without relying on expensive and complicated instrumentations to support the in situ sensing, fast processing of state estimation and implementation of control algorithms. A regression metamodel of heat transfer, combined with an auxiliary thermal model, was used to generate the training data for a model-free optimal controller, which adjusted the laser scan speed to achieve various temperature-based control objectives in a numerical study of L-PBF and DED processes [25]. In [26], laser power was controlled based on the locally varying relative proportion of solid or powder material to improve the surface quality of a part built with L-PBF. A feedforward controller of laser power was developed based on an analytical model of melt-pool cross-sectional area to improve the build quality, with experimental validation on an EOSINT M280 L-PBF AM system [27]. In [28], computational approaches were developed to identify L-PBF scan vectors that require process parameter adaptation and then a simulation-based feedforward control was applied to optimize the complex geometries. A model-based adaptive control of laser power was applied in terms of the temperature region to ensure various build-quality metrics in laser welding and laser-based AM [29].

In this paper, based on our prior model on the dynamics of melt-pool height [30], we develop a nonlinear inverse-dynamics controller to modulate the laser power in a hatch-by-hatch, layer-by-layer manner such that the melt-pool height in each deposition vector is regulated to its target value. L-shaped structures consisting of an one-bead leg and a three-bead leg, which have been considered in the experimental study of thermal characteristics and microstructure for DED [31,32], are used in this paper as a case study to illustrate the control design. The trajectory of the laser power, obtained from the simulated closed-loop control, is implemented as a feedforward control in depositing the L-shaped structure on an Optomec LENS MR-7 system. The produced average height error is then compared to the average height error when a constant laser power is applied throughout the entire build process, as well as to the height error when a hatch-dependent constant laser power was applied based on experimental trial and error.

Compared to the work in [30], which is a modeling paper, the contribution of the current paper lies in the development of a nonlinear inverse-dynamics controller to control the part height and experimental validation of the proposed controller on the Optomec LENS MR-7 system. Preliminary work of this paper was presented in the ASME Dynamic Systems and Control Conference [33], which introduced the concept of the proposed controller and provided some primitive results. The current paper has added significant analysis and experimental results. The figures and tables of data on simulation and experimental results are new in this manuscript.

## 2. Dynamic Model on Melt-Pool Height

This section gives a brief overview of our prior lumped-parameter model on melt-pool height dynamics for DED [16,30], for which this paper considers the special case that the laser power Q(t) is the only varying process parameter while keeping the laser scan speed *v* and the powder flow rate *f* fixed.

### 2.1. Single-Hatch Deposition

For a single-hatch deposition, in terms of the melt-pool mass conservation and an approximation of the material transfer rate (the product of the powder catchment efficiency μ and the powder flow rate *f*, i.e., μf) derived in [16], the melt-pool volume V(t) and cross-sectional area A(t) satisfy the following lumped-parameter model [16],

(1) $$\rho \frac{dV(t)}{dt}=-\rho A\left(t\right)v+\beta \frac{\eta (Q\left(t\right)-Q^{c})}{\pi c_{l}\left(T_{m}-T_{initial}\right)},\;Q>Q^{c}$$

where ρ denotes the melt density, η denotes the laser transfer efficiency, $c_{l}$ is the molten material specific heat, $T_{m}$ is the melting temperature of the material, $T_{initial}$ denotes the initial temperature of the workpiece, β is a constant linear coefficient, and $Q^{c}$ denotes a critical value of the laser power, for which the melt-pool volume equals zero if the laser power is less than this threshold value.

Further, assume that the melt-pool takes the shape of a half ellipsoid with fixed aspect ratios [16], where the ratio of melt-pool width over melt-pool length *l* is assumed to be one, i.e., w/l=1. In Table 4 of the work in [31], measurements from the coaxial ThermaViz^®^ images of melt-pools of the L-shaped builds showed that the melt-pool length-to-width ratio varied within [0.873, 1.085] at different build locations, and thus a length-to-width ratio of w/l=1 is adopted here. Further, let *r* denote the constant ratio of melt-pool width *w* over melt-pool height *h*, i.e., r=w/h. Then, the melt-pool height satisfies [16]

(2) $$\frac{\pi }{2}\rho r^{2}h^{2}\left(t\right)\frac{dh(t)}{dt}=-\frac{\pi }{4}\rho rh^{2}\left(t\right)v\left(t\right)+\beta \frac{\eta (Q\left(t\right)-Q^{c})}{\pi c_{l}\left(T_{m}-T_{initial}\right)},\;Q>Q^{c}$$

### 2.2. Multi-Hatch Deposition

The dynamic equation of melt-pool height for a single-hatch deposition in Equation (2) was then extended to the case of multi-hatch (and multi-layer) deposition in [30], by recomputing the initial temperature $T_{initial}$ to account for the thermal history in the build process. When a build consists of more than one hatch, or of single-hatch but with more than a single layer (single-hatch walls), the initial temperature $T_{initial}$ in Equation (2) is no longer equal to the ambient temperature or the preheated substrate temperature, rather, $T_{initial}$ needs to account for the thermal history in the build process.

**Figure 1:**
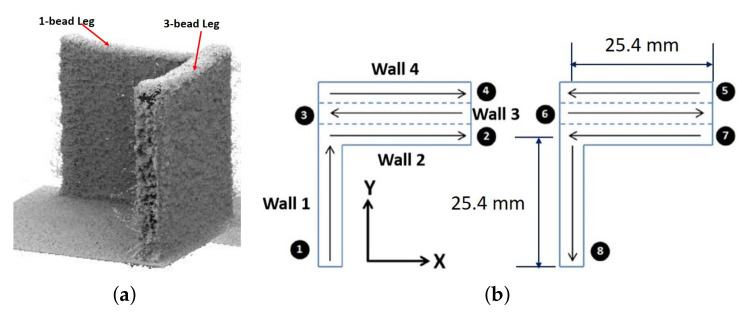
(**a**) L-shaped structure consisting of an 1-bead leg and a 3-bead leg. (**b**) Eight-hatch build plan of the L-shaped structure, where hatches 1–4 are for odd layers and hatches 5–8 are for even layers. *Wall 1*: single-bead vertical wall built by hatch 1 and hatch 8; *Wall 2*: vertical component of the 3-bead leg built by hatch 2 and hatch 7; *Wall 3*: vertical component built by hatch 3 and hatch 6; *Wall 4*: vertical component built by hatch 4 and hatch 5.

Consider a L-shaped structure consisting of an one-bead leg and a three-bead leg, as illustrated in Figure 1a,b, which shows its eight-hatch build path, where Hatches 1–4 correspond to the deposition path in odd layers and Hatches 5–8 are for the even layers. For the L-shaped structure, the initial temperature of a point of interest at coordinate (x,y,z) before laser deposition is derived as follows [30],

(3) $$T_{initial}\left(x,y,z\right)=T_{0}+\Delta T_{initial}^{X}\left(x,y,z\right)+\Delta T_{initial}^{Y}\left(x,y,z\right)$$

where $T_{0}$ denotes the initial temperature of the substrate before deposition, $\Delta T_{initial}^{X}$ and $\Delta T_{initial}^{Y}$ denote the temperature contributions from the past hatches deposited along the *X* direction (the set of *past* hatches 2–7, denoted by $H_{X}$), and the temperature contributions from the past hatches deposited along the *Y* direction (the set of *past* hatches 1 and 8, denoted by $H_{Y}$). Corresponding to each past hatch $i,i\in H_{X}$ or $i\in H_{Y}$, a virtual heat source *i* is assigned to replace the original laser heat source as soon as it finishes depositing hatch *i*. The virtual heat source *i* has the same power as the original laser heat source depositing the hatch *i* and continues to travel with the same speed along the same direction. Consider that the laser heat source deposits the hatch *i* with a net power $q_{i}$ (for a laser power $Q_{i}$ with laser efficiency η, $q_{i}=\eta Q_{i}$) and scanning speed $v_{i}$, and let $\left(x_{i},y_{i},z_{i}\right)$ denote the coordinate of the virtual heat source *i*, then

(4) $$\begin{array}{ccc} \Delta T_{initial}^{X}\left(x,y,z\right) & = & \sum_{i\in H_{X}}\frac{q_{i}}{2\pi kR_{i}}e^{\frac{-v_{i}\left(w_{i}^{x}+R_{i}\right)}{2a}} \end{array}$$

(5) $$\begin{array}{ccc} \Delta T_{initial}^{Y}\left(x,y,z\right) & = & \sum_{i\in H_{Y}}\frac{q_{i}}{2\pi kR_{i}}e^{\frac{-v_{i}\left(w_{i}^{y}+R_{i}\right)}{2a}} \end{array}$$

where the temperature contribution from each past hatch *i*, $\frac{q_{i}}{2\pi kR_{i}}e^{\frac{-v_{i}\left(w_{i}^{y}+R_{i}\right)}{2a}}$, is computed in terms of the Rosenthal’s solution [34] induced from the virtual heat source *i*; further, $w_{i}^{x}=x-x_{i}$, $w_{i}^{y}=y-y_{i}$, *k* denotes the thermal conductivity, *a* denotes the thermal diffusivity, and $R_{i}$ denotes the distance from (x,y,z) to $\left(x_{i},y_{i},z_{i}\right)$. Similar to the virtual heat sources here, mirrored virtual heat sources could be defined for an insulated boundary condition on a plane. Curved hatches could not be accommodated by the Rosenthal solution.

### 2.3. Moving-Source Model in Space Coordinate

Consider the moving-source model with a constant laser scan speed *v*, and let *s* denote the deposition distance from the start of the hatch along the deposition direction. By applying the following relationship,

(6) $$\frac{dh}{dt}=\frac{dh}{ds}\times \frac{ds}{dt}\Rightarrow \frac{dh}{ds}=\frac{1}{v}\times \frac{dh}{dt}$$

the height dynamics in Equation (2) can then be converted into an equation in the space domain with respect to *s*:

(7) $$\frac{dh(s)}{ds}=\frac{2\beta \eta }{\rho \pi^{2}r^{2}c_{l}v\left(T_{m}-T_{initial}\left(s\right)\right)h^{2}\left(s\right)}\left(Q\left(s\right)-Q^{c}\right)-\frac{1}{2r},\;Q>Q^{c}$$

For a part consisting of multiple hatches and multiple layers, the melt-pool height h(s), laser power Q(s), and the initial temperature $T_{initial}\left(s\right)$ are defined as layer- and hatch-specific. For a given *s* in a specific layer or hatch, the corresponding coordinates (x,y,z) can be determined easily from the build plan, and then Equations (3)–(5) can be applied to compute $T_{initial}\left(s\right)$. As a result, Equation (7) shows that the melt-pool height dynamics at different hatches (and/or different layers) only differentiate in the initial temperature $T_{initial}\left(s\right)$, and such $T_{initial}\left(s\right)$ can be precalculated before depositing the current hatch since it is only affected by the past hatches.

In this paper, prediction of the part height of the DED build is computed by adding up the melt-pool height over all deposited layers, by ignoring any possible overlap of melt-pool height across two adjacent layers. For laser clad depositions, a two-dimensional (2D) cross-sectional model was developed in [35] to predict the relative coating height in multi-track, multi-layer depositions as a function of hatch overlap ratio, where the relative coating height was defined as the height of the coating measured from the substrate normalized by the height of an individual track. The hatch overlap ratio is defined as $OR=(w-s_{h})/w$ with *w* denoting the melt-pool width and $s_{h}$ denoting the hatch spacing between two individual tracks. It was shown that for the overlap ratio of 30%, the relative coating height for 2 layers, 4 layers, and 6 layers was approximately 2, 4, and 6, respectively (see Figure 3 of [35]). The model prediction was validated by experimental measurements from depositions of multi-track multi-layer of stainless steel when the effect of thermal history was negligible due to a sufficient long inter-layer dwell time (enough time given to the first layer to cool down before applying the second layer) (see Figure 10 of [35]). In this paper, the hatch spacing is selected for a 33% hatch overlap ratio (more details given in Section 4), and thus, based on the study of [35], it is assumed that the melt-pool height equals to the layer height, by ignoring any possible melt-pool height overlap across two adjacent layers.

## 3. Control Design

### 3.1. Control Problem Formulation

The control problem is formulated as follows. Assume that the laser power *Q* in deposition is the only control variable, with other process parameters fixed. The control objective is to design Q(s) in each deposition vector (hatch) such that a target part height $H_{r}$ is achieved at the end of the build process.

Now consider the L-shaped structure and its build plan shown in Figure 1. As labeled in Figure 1b, *Wall 1* denotes the 1-bead leg built by hatches 1 and 8; *Wall 2* by hatches 2 and 7; *Wall 3* by hatches 3 and 6; and *Wall 4* by hatches 4 and 5. That is, the 3-bead leg consists of three vertical components—*Wall 2*, *Wall 3*, and *Wall 4*—joining together. Let $H_{r}$ denote the target part height, and let *N* denote the total number of layers of the part. In an ideal situation, the deposition of each layer will render the same melt-pool height, e.g., equal to $\bar{h}=H_{r}/N$ if ignoring any overlap of melt-pool height across two adjacent layers. When the target melt-pool height $\bar{h}$ is not met in one hatch in a layer, the reference melt-pool height in the next layer needs to be adjusted so that the height of the final build will reach $H_{r}$. Therefore, we define the reference height of the melt-pool at each hatch indexed by (layer *i*, wall *j*) for any i∈{1,…,N} and j∈{1,2,3,4} as follows,

(8) $$h_{i,j}^{r}\left(s\right)=\left\{\begin{array}{cc} \bar{h} & \left(i=1\right)\\ i\times \bar{h}-\sum_{m=1}^{i-1}h_{m,j}\left(s\right) & \left(i=2,\ldots ,N\right) \end{array}\right.$$

where $h_{m,j}$ denotes the melt-pool height of the hatch in (layer *m*, wall *j*), then $\sum_{m=1}^{i-1}h_{m,j}\left(s\right)$ corresponds to the height of the existing *wall j* before depositing the *i*th layer, which is computed by adding up the melt-pool height from layer *1* to layer (i−1) of *wall j* and ignoring any possible overlap of melt-pool height across two adjacent layers.

To this end, the control objective is reduced to designing $Q_{i,j}\left(s\right)$ for each hatch in (layer *i*, wall *j*) in a hatch-by-hatch, layer-by-layer manner such that the resulting melt-pool height achieves the reference value $h_{i,j}^{r}\left(s\right)$.

### 3.2. Control Design for Laser Power in a Hatch-by-Hatch, Layer-by-Layer Manner

Next, consider each hatch indexed by (layer *i*, wall *j*), we design the laser power $Q_{i,j}\left(s\right)$ so that the melt-pool height $h_{i,j}\left(s\right)$ of the hatch at (layer *i*, wall *j*) will reach $h_{i,j}^{r}\left(s\right)$ for s∈[0,L], where *L* denotes the length of the hatch (or $L_{i,j}$ if hatches have different length).

Note that the laser power *Q* varies along the deposition distance *s*. In applying Equations (4) and (5) to compute $\Delta T_{initial}^{X}$ and $\Delta T_{initial}^{Y}$ due to all past hatches, the power of any virtual heat source representing a past hatch is assumed to take the value of the physical laser power applied at the end of that past hatch, i.e., at s=L.

Consider each hatch indexed by (layer *i*, wall *j*), for the simplicity of illustration, we use short-hand notations $h^{r}\left(s\right)$ and h(s) to represent $h_{i,j}^{r}\left(s\right)$ and $h_{i,j}$, respectively, and further drop the subscript (i,j) for later notations. Then, define the tracking error in melt-pool height as

(9) $$e\left(s\right)=h\left(s\right)-h^{r}\left(s\right)$$

Further define F(s,h(s)):

(10) $$F\left(s,h\left(s\right)\right)=\frac{2\beta \eta }{\rho \pi^{2}r^{2}c_{l}v\left(T_{m}-T_{initial}\left(s\right)\right)h^{2}\left(s\right)}$$

and define

(11) $$\tilde{Q}\left(s\right)=Q\left(s\right)-Q^{c}$$

Then, by Equation (7) and Equations (9)–(11), the error dynamics at each hatch satisfy

(12) $$\frac{de(s)}{ds}=\frac{dh(s)}{ds}-\frac{dh^{r}\left(s\right)}{ds}=F\left(s,h\left(s\right)\right)\tilde{Q}\left(s\right)-\frac{1}{2r}-\frac{dh^{r}\left(s\right)}{ds}$$

Define a new control input $\hat{Q}\left(s\right)$ such that the error dynamics of the melt-pool height satisfy

(13) $$\frac{de(s)}{ds}=\hat{Q}\left(s\right)$$

and further consider a simple proportional control for $\hat{Q}\left(s\right)$ as follows,

(14) $$\hat{Q}\left(s\right)=-K_{p}e\left(s\right)$$

where $K_{p}>0$ denotes the proportional control gain. Then, we have

(15) $$\frac{de(s)}{ds}=-K_{p}e\left(s\right)$$

This leads to stable error dynamics for any $K_{p}>0$, where the error in achieving the reference melt-pool height converges to zero with an exponential rate of $e^{-K_{p}s}$.

By Equations (9), (11), (12) and (15), the control input for laser power Q(s) is derived as follows,

(16) $$\begin{array}{ccc} Q(s) & = & Q^{c}+\frac{1}{F(s,h(s))}\left\{-K_{p}\left[h\left(s\right)-h^{r}\left(s\right)\right]+\frac{dh^{r}\left(s\right)}{ds}\frac{1}{2r}\right\}\\ & = & Q^{c}+\frac{\rho \pi^{2}r^{2}c_{l}v\left(T_{m}-T_{initial}\left(s\right)\right)h^{2}\left(s\right)}{2\beta \eta }\left\{-K_{p}\left[h\left(s\right)-h^{r}\left(s\right)\right]+\frac{dh^{r}\left(s\right)}{ds}\frac{1}{2r}\right\} \end{array}$$

which is a nonlinear inverse-dynamics controller requiring real-time feedback of the melt-pool height h(s). Such laser power control has to be performed in a hatch-by-hatch, layer-by-layer manner following the sequence of the build plan given in Figure 1b, as the reference melt-pool height $h^{r}\left(s\right)$ and the initial temperature $T_{initial}\left(s\right)$ need to be updated hatch-by hatch, and layer-by-layer.

## 4. Simulation Results

Consider the L-shaped structure of Ti-6AL-4V shown in Figure 1a, which has 143 layers and consists of an 1-bead leg and a 3-bead leg. The part is built on a substrate of Ti-6AL-4V with the dimensions of 76.2 mm × 76.2 mm × 6.35 mm, by following the eight-hatch build plan shown in Figure 1b, where hatches 1–4 are for odd layers and hatches 5–8 are for even layers. Each hatch has a length of 25.4 mm. Following the definition of the X and Y directions shown in Figure 1b, Hatch 1 is from (*x* = 0, *y* = 0) to (*x* = 0, *y* = 25.4 mm), and without pausing, hatch 2 goes from (0, 25.4 mm) to (25.4, 25.4) mm, then after a jump, hatch 3 goes from (25.4, 25.4 + 0.8128) mm to (0, 25.4 + 0.8128) mm, and after another jump, hatch 4 goes from (0, 25.4 + (2 × 0.8128)) mm to (25.4, 25.4 + (2 × 0.8128)) mm. The scanning speed *v* of the laser is kept at a constant value of v=10.58 mm/s, and the powder flow rate is kept at *f* = 3 g/min = $5\times 10^{-5}$ kg/s, through the entire build process. The hatch spacing of $s_{h}=0.8128$ mm here is designed to be 0.67 of the expected melt-pool width *w* for a 33% overlap in melt-pool width between two adjacent hatches, where the overlap ratio is defined as $OR=(w-s_{h})/w$. There is no additional inter-hatch dwell time except the time required to move the laser head across the hatch spacing of 0.8128 mm.

### 4.1. Open-Loop Simulation for Part Height

**Figure 2:**
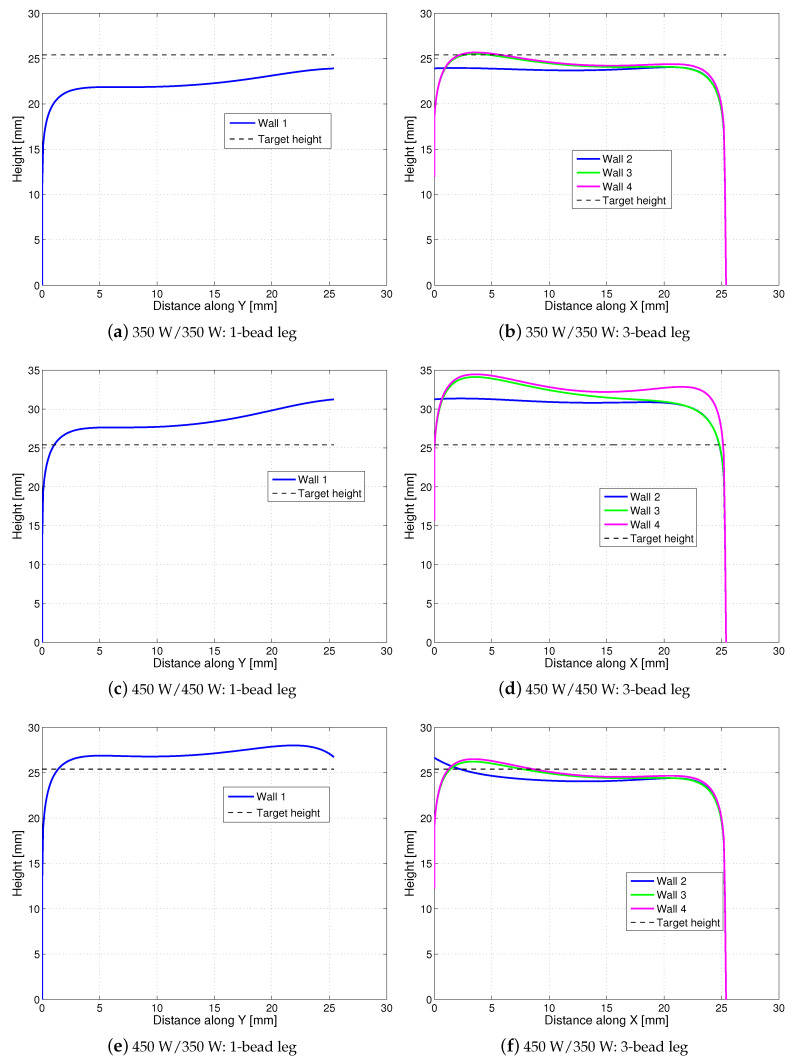
Open-loop simulation for part height under three constant laser-power strategies: (**a**,**b**) 350 W/ 350 W, (**c**,**d**) 450 W/ 450 W, and (**e**,**f**) hatch-dependent 450 W/350 W. The dashed line in each subplot represents the target build height of 25.4 mm.

The simulation parameters are given in Table 1, where the thermal conductivity and thermal diffusivity are assumed to be constant rather than temperature-dependent in computing the initial temperature as the Rosenthal’s solution is utilized. Figure 2 plots the simulated part height under three different laser power strategies: (1) a constant laser power of 350 W through the entire build, (2) a constant laser power of 450 W through the entire build, and (3) hatch-dependent laser power, where 450 W is applied to the 1-bead leg and 350 W is applied to the 3-bead leg. The laser power values tested here, such as 350 W and 450 W, were chosen based on experimental trial and error with the objective of achieving a target build height of 1 inch (25.4 mm) with two legs having similar height. The height of each wall (*Wall 1–4*) is computed by summing up the melt-pool height predicted by Equation (7) over all layers and ignoring any possible overlap of melt-pool height across two adjacent layers.

Table 2 summarizes the corresponding average leg height under each laser-power strategy, where the average height of the 3-bead leg is computed as follows. First, at each distance *s* along the *X*-direction, compute the maximum wall height among *Walls 2–4*, and then compute the average of them over the distance *s*. It can be seen that under 350 W/ 350 W, the height of each leg is lower than the target part height of 25.4 mm, especially the 1-bead leg is 12.6% lower than the target value even without considering the overlap of melt-pools across two adjacent layers. Under the laser power strategy of 450 W/450 W, the height of each leg is about 11.4–28.74% higher than the target part height, and the 3-bead leg is about 16% higher than the 1-bead leg, due to more thermal build-up during depositing three adjacent hatches than depositing a single hatch. Among these three laser power strategies here, the hatch-dependent 450 W/350 W achieves the best performance, with the predicted average height within 6% of the target build height.

### 4.2. Closed-Loop Simulation under Nonlinear Inverse-Dynamics Control

This section shows the simulation results of the closed-loop control, based on the melt-pool height dynamics in Equation (7), the targeted melt-pool height defined in Equation (8), and the nonlinear inverse-dynamics controller in Equation (16). Simulation parameters are given in Table 1. A lower bound of 250 W and an upper bound of 425 W are set for the laser power to reflect the process limit. A slightly tighter upper bound than 450 W is applied here and later simulation results show that such upper bound is touched mainly to compensate for the tapered-off ends of the L-shaped build.

**Figure 3:**
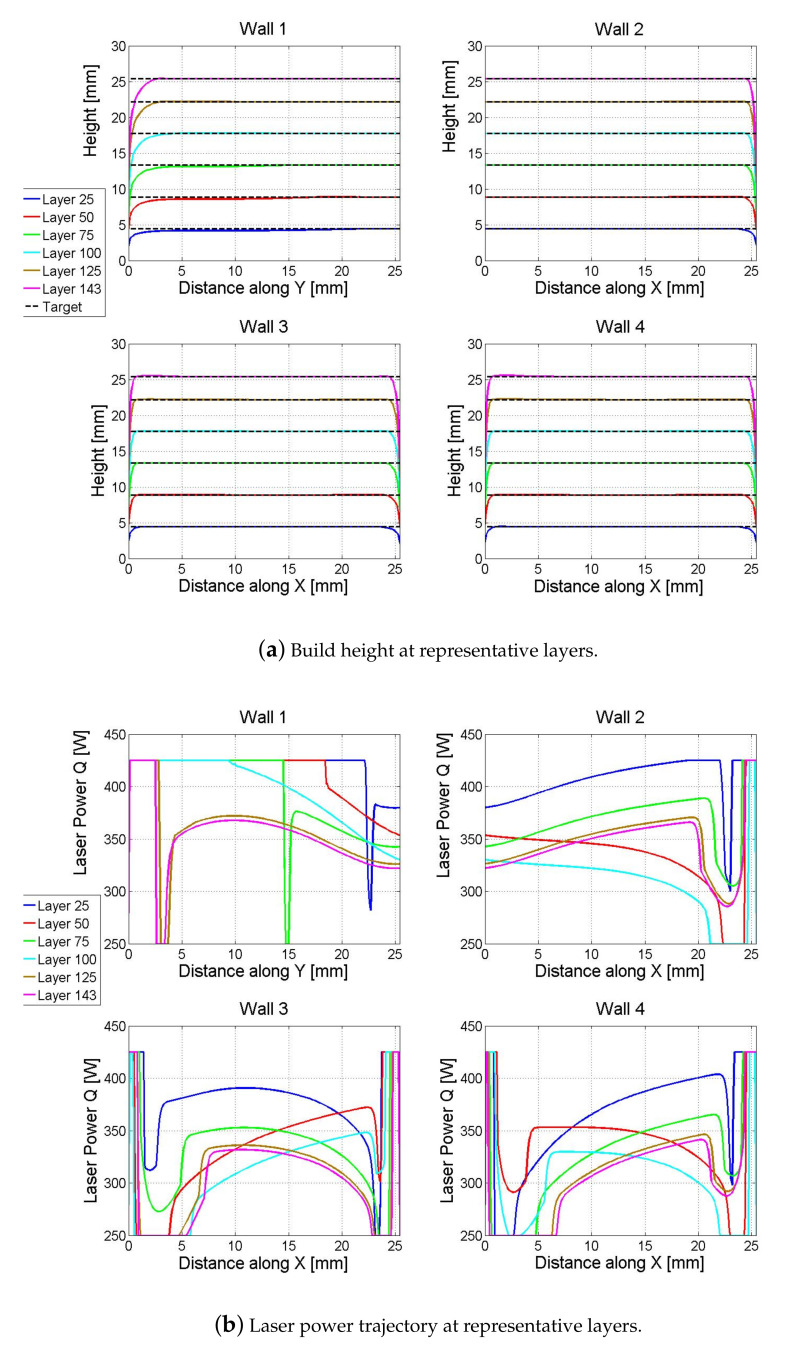
Closed-loop simulation under the nonlinear inverse-dynamics control. (**a**) Build height at representative layers. (**b**) Laser power trajectory at representative layers.

**Figure 4:**
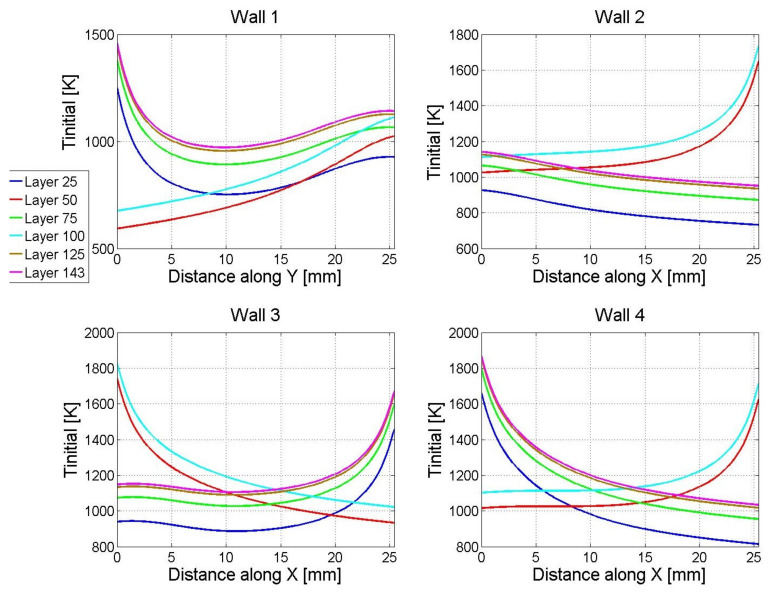
Initial temperature $T_{initial}$ (K) at Layer 25, 50, 75, 100, 125, and 143 during the nonlinear inverse-dynamics control.

The closed-loop simulation is illustrated in Figure 3 and Figure 4. Figure 3a shows the build height of the L-shaped structure after depositing a number of representative layers, e.g., after depositing 25, 50, 75, 100, 125, and 143 layers, and Figure 3b shows the corresponding laser power trajectories at the representative layers. Figure 4 shows the simulated initial temperature during the nonlinear control. The build height of each wall is computed by adding up the melt-pool height of the corresponding hatches over all deposited layers. It can be seen that the target build height is achieved after 143 layers except at the end edges of the walls, where the wall height tapers off at ends (around distance of 0 mm or 25 mm).

As expected, at the same representative layers, the laser powers applied in the 3-bead leg (*Wall 2–Wall 4*) are generally lower than that applied in the 1-bead leg, as if the same laser power was applied for both legs, the build heights would differ significantly between the two legs due to more thermal build-up in the three-bead deposition than in the 1-bead deposition.

For the 1-bead leg (*Wall 1*), although the laser powers at the lower layers (Layer 25 and Layer 50) touch the upper bound during more than half of the deposition, the laser powers at the higher layers (Layer 125 and Layer 143) are lower than the upper bound for most of the deposition (except at the ends of the hatches) due to heat build-up over time as illustrated by the initial temperature during the simulated deposition of *Wall 1* in Figure 4.

For the 3-bead leg, without loss of generality, take the laser power trajectory at Layer 25 of *Wall 4* as an example, which corresponds to hatch 4 in the build path as it is at an odd layer. It can be seen that the laser power starts at the upper bound to compensate for the tapered-off height at wall end due to the initial transient (e.g., at X-distance of 0–2 mm of *Wall 4* shown in Figure 2f). Then, the laser power decreases to its lower bound since the temperature at this area is still very high due to that the laser has just swept through to finish depositing hatch 3 (see the initial temperature shown in Figure 4). The heat accumulation would cause over-built if no modulation of laser power was applied, e.g., the over-built hump at X-distance of 2–5 mm of *Wall 4* under the constant laser power shown in Figure 2f. With the increase of the distance along the *X*-direction, the thermal contribution from depositing hatch 3 is reduced (i.e., the area ahead of the laser gets cooler) and thus the laser power is increased accordingly.

Similarly, at Layer 50 of *Wall 4*, which corresponds to hatch 5 in the build path as it is at an even layer, the laser starts deposition from *X* = 25.4 mm at the highest power (upper bound) to compensate for the tapered-off height at the wall end, and then it moves towards the −*X* direction. Due to the deposition of hatch 4 in the previous layer, the area close to *X* = 25.4 mm is still very hot, and thus the corresponding laser power hits the lower bound. With the laser moving towards the −*X* direction, the laser power gradually increases to compensate for the cooler temperature in the area ahead of the laser. If a higher upper bound of the laser power was applied, it might slightly improve the height compensation at the tapered-off end of the L-shaped structure, but not likely to affect much for the rest of the build.

## 5. Experimental Methods

**Figure 5:**
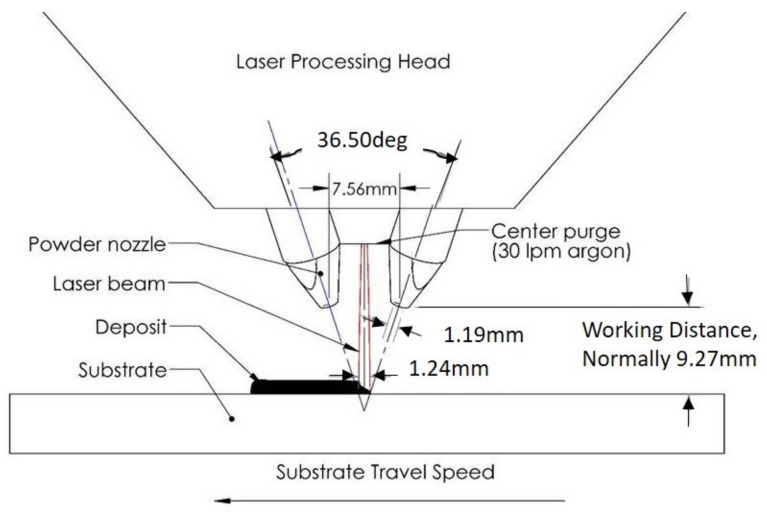
A schematic plot of the Laser-Engineered Net Shape (LENS) process used in experimental validation.

To experimentally evaluate the proposed model-based control design for laser power, a number of L-shaped structures of Ti-6AL-4V were built with the Optomec LENS MR-7 system using a 500 W, 1070 nm wavelength IPG photonics fiber laser. A schematic plot of the Optomec MR-7 system is given in Figure 5. Three laser-power strategies were examined: (i) Constant power (*C*): a constant laser power of 450 W was applied through the entire build; (2) Hatch-dependent, constant power (*HD-C*): a constant laser power of 450 W was applied to the 1-hatch (1-bead) leg, whereas a constant laser power of 350 W was applied to the 3-hatch (3-bead) leg of the L-shaped structure; and (3) Model-based feedforward control (*MB-FF*): the laser power trajectories shown in Figure 3b from the simulated closed-loop control were then implemented as a feedforward controller to vary laser power in depositing the L-shaped structure. Two independent samples were built under each laser-power strategy. Other process parameters such as the laser scanning speed, hatch spacing, powder flow rate, substrate temperature, inter-hatch dwell time, length of each hatch, and number of layers in each build take the same values as given in Table 1.

**Figure 6:**
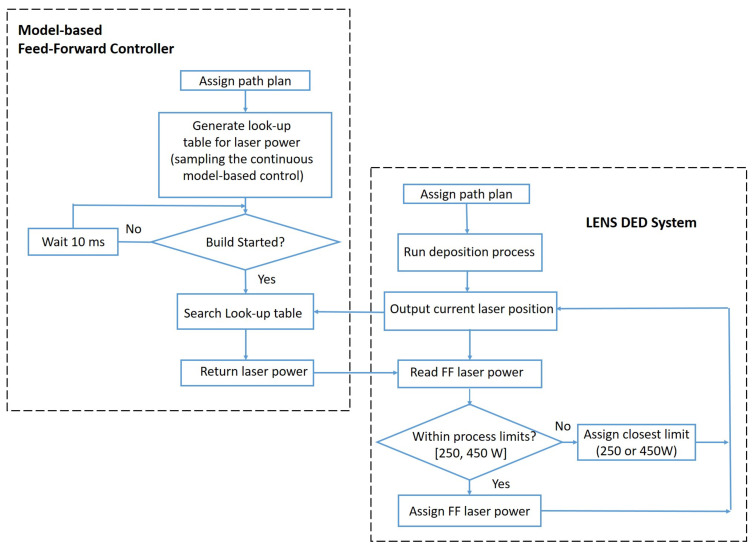
Flow chart of implementing the model-based feedforward (MB-FF) controller for laser power in the LENS Directed Energy Deposition (DED) system.

In implementing the model-based feedforward control using the laser power trajectory from Figure 3b, the continuous laser power trajectory Q(s) for each hatch in each layer was sampled with an equal space of Δs=0.1 mm. Corresponding to the length of each hatch *L* = 25.4 mm, this led to a total of 255 samples for the laser to deposit each hatch. Then, the sequence of laser powers together with their respective coordinates of the laser position were assembled into a lookup table for laser power to deposit the L-shaped structure. Figure 6 shows a flow chart on how the *MB-FF* controller was implemented in the LENS MR-7 system. After the path plan was assigned, the laser position and the associated laser power were first read from the preloaded lookup table, and then the laser power was set accordingly if it was within the process limit, or reset to its lower- or upper-bound otherwise.

## 6. Results of Experimental Evaluation

### 6.1. Measured Height of Experimental Samples

**Figure 7:**
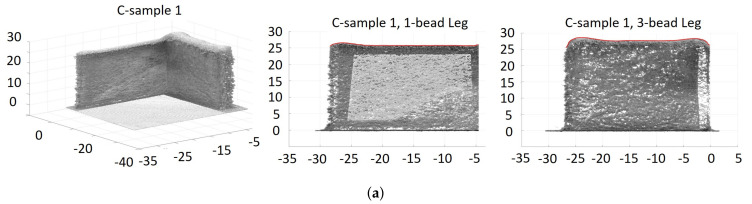

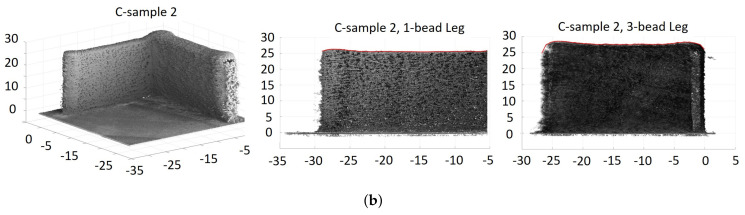
Scatter plots of point cloud data from coordinate measuring machine (CMM) images of the L-shaped samples built with the constant laser power of 450 W, where each red solid line represents the top envelop of each leg. (**a**) Sample 1. (**b**) Sample 2.

**Figure 8:**
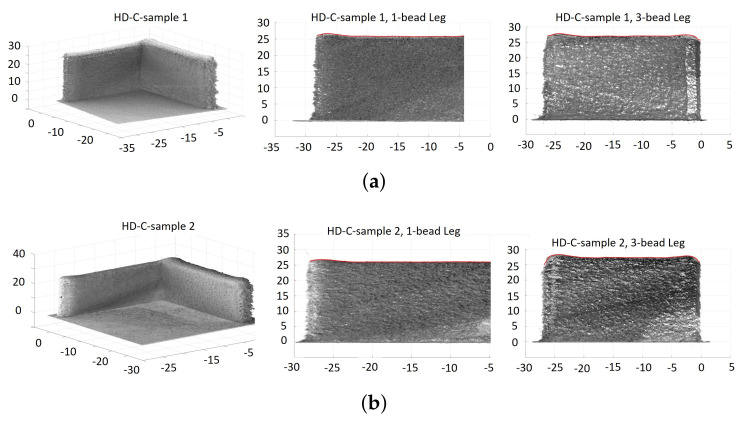
Scatter plots of point cloud data from CMM images of the L-shaped samples built with the hatch-dependent laser power of 450 W/350 W, where each red solid line represents the top envelop of each leg. (**a**) Sample 1. (**b**) Sample 2.

**Figure 9:**
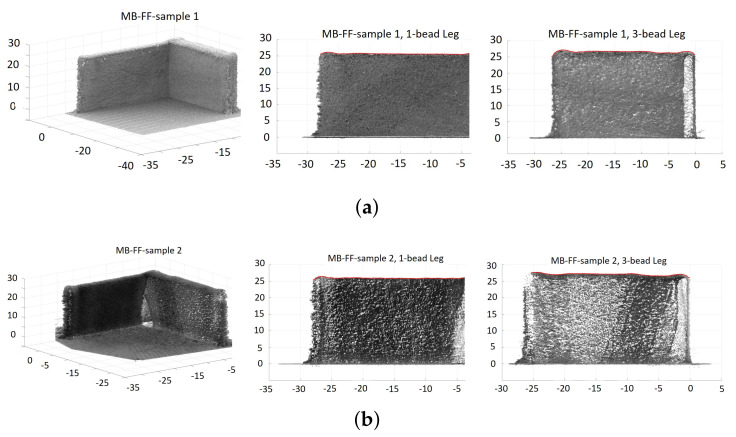
Scatter plots of point cloud data from CMM images of the L-shaped samples built with the MB-FF control of laser power, where each red solid line represents the top envelop of each leg. (**a**) Sample 1. (**b**) Sample 2.

As described in Section 5, two L-shaped samples were built under each of the three laser-power strategies: *C*, *HD-C*, and *MB-FF*. Image scanning of these samples was performed through a FARO Edge coordinate measuring machine (CMM) (2.7 m, seven axis) coupled with a FARO Laser Line Probe ES, with ±0.041 mm volumetric accuracy [36]. Raw imaging data from the CMMwere then processed through rotation, shifting, and slicing to obtain the corresponding image and part height of each leg of the L-shaped samples. Figure Figure 7, Figure 8 and Figure 9 show the scatter plots of point cloud data from the CMM images of the L-shaped samples, including the entire build and each of the two legs, under the laser power strategy *C*, *HD-C*, and *MB-FF*, respectively.

**Figure 10:**
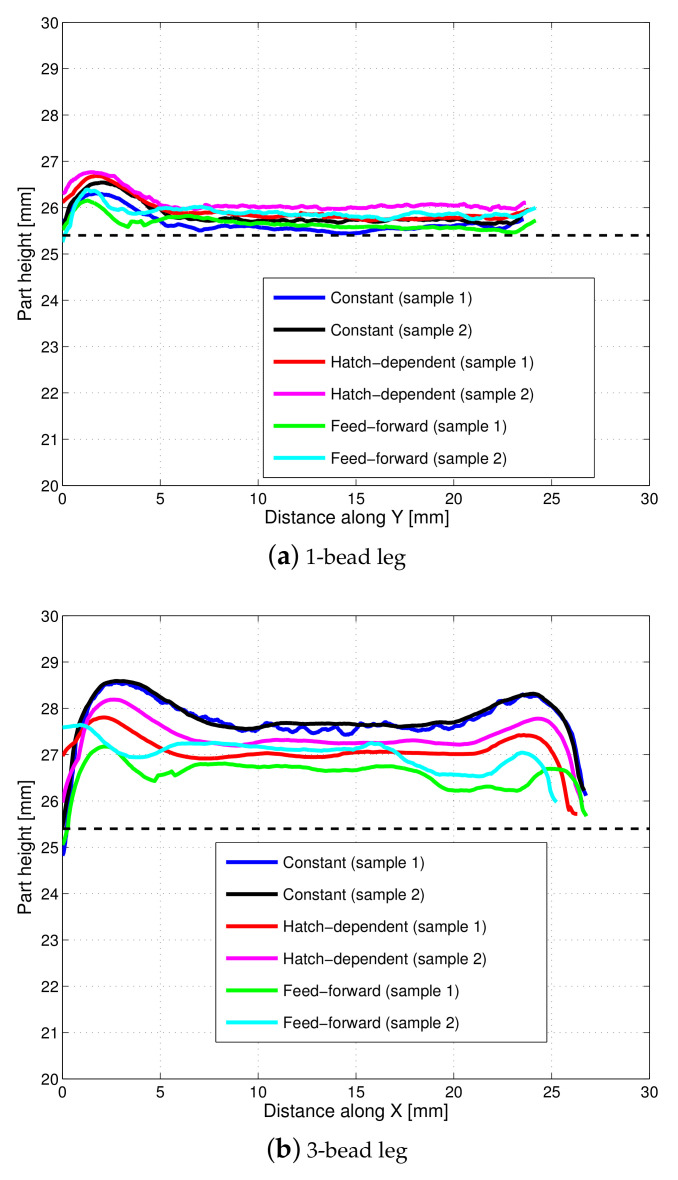
Build height comparison among the three laser power strategies: (1) Constant (450 W), (2) hatch-dependent (450 W/350 W), and (3) model-based feedforward control. Two samples were fabricated under each laser power strategy. Each line represents the top envelop of each leg of the L-shaped samples. (**a**) 1-bead leg. (**b**) 3-bead leg.

To illustrate the build height of the six samples (two from each laser power strategy), Figure 10 plots the top envelop line of each leg from each of the six L-shaped samples, noting that each top envelop is represented by a red solid line to trace the part height of each sample leg in Figure 7, Figure 8 and Figure 9. It is observed from Figure 10 that for the 1-bead leg, all samples have very similar part height and it is close to the target height of 25.4 mm. However, the height of the 3-bead leg varies among different laser power strategies. Despite of sample variance, the height of the 3-bead leg of the samples built with *MB-FF* is the closest to the target part height of 25.4 mm, whereas the part height of the samples built with *C* shows the most deviation from the target value. Nevertheless, the top surfaces of the samples built with *MB-FF* appear to have the most unevenness. The higher variability of part height under *MB-FF* was attributed to both the modeling errors and lack of a feed-back correction. The measured average height (and standard deviation) of each leg of the L-shaped samples is given in Table 3.

### 6.2. Model Validation

To validate the open-loop simulation of part height, the model-predicted values in Table 2 under the laser power 450 W/450W (*C*) and 450 W/350W (*HD-C*) are compared to the experimental measurements for *C* and *HD-C* given in Table 3. It is shown that for the laser power strategy of 450 W/450 W, the model has overpredicted the height by 9.7% for the 1-bead leg and by 17.7% for the 3-bead leg. Under 450 W/350 W, the model has overpredicted the height by 3.3% for the 1-bead leg and underpredicted by 8.5% for the 3-bead leg. The comparison of the predicted part height in Figure 2c–f to the measured part height in Figure 10 demonstrates the same trend.

It is worth pointing out that since titanium β grains grow epitaxy from the underlying substrate upon solidification, then transition to an α+β microstructure, it is not possible to identify where one layer has started and the new layer has formed from the Ti-6AL-4V DED builds. One can typically observe reheating bands, partially aligned grains (colony α) that look like layer boundaries, but they should not be confused with the actual layer boundaries. For the same reason, the melt-pool boundaries within the Ti-6AL-4V DED builds cannot be identified either, as demonstrated by Figure 3 of [37] on a polished and etched cross section of Ti-6AL-4V build with an overlay of expected hatch locations. Therefore, the hatch overlap ratio, as well as the actual melt-pool height versus the layer height in this paper, cannot be experimentally validated.

### 6.3. Evaluation of the Performance of MB-FF

To quantify the performance of regulating the build height to its target value, the average normalized error e% between the measured and targeted build height, and the root mean square error (RMSE) are defined as

(17) $$\begin{array}{ccc} e\% & = & \frac{\sum_{i=1}^{n}\left|H_{m}\left(i\right)-H_{r}\right|/H_{r}}{n}\times 100\% \end{array}$$

(18) $$\begin{array}{ccc} RMSE & = & \sqrt{\frac{\sum_{i=1}^{n}{\left(H_{m}\left(i\right)-H_{r}\right)}^{2}}{n}} \end{array}$$

where $H_{m}\left(i\right)$ denotes the measured build height at a sample distance *i* along the deposition direction, and $H_{r}=25.4$ mm denoting the target build height. Such average error e% and *RMSE* are calculated for each leg of each L-shaped sample structure. Note that using the point cloud data from the FARO CMM for each leg of each L-shaped sample structure, a top envelop line is generated (see the red solid line defining the build height of each leg in Figure 7, Figure 8 and Figure 9). The sample measurements on this top envelop are used to compute the average error e% and *RMSE*. Table 3 summarizes the average error and *RMSE* of the part height of each L-shaped structure built by *C*, *HD-C*, and *MB-FF*.

Consistent with the observations from Figure 10, Table 3 shows that the build height of the 1-bead leg is achieved with negligible error (less than 3% error) under each of the three laser power strategies. Overbuild is observed for the 3-bead leg under all laser-power strategies, with *C* having the highest overbuild (9–10% higher than the target height) and *MB-FF* having the lowest overbuild (5–6% higher than the target height). Overall, for the 3-bead leg, the *MB-FF* is able to reduce the average error rate of build height by 42% compared to *C* and by 24% compared to *HD-C*. This result indicates that *MB-FF* could be a viable solution for build height control in a DED process to replace the experimental trial and error, especially when the real-time, measurement feedback control is not available or too expensive/difficult to implement.

**Figure 11:**
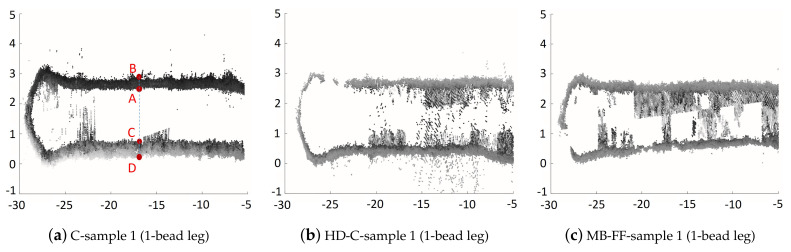

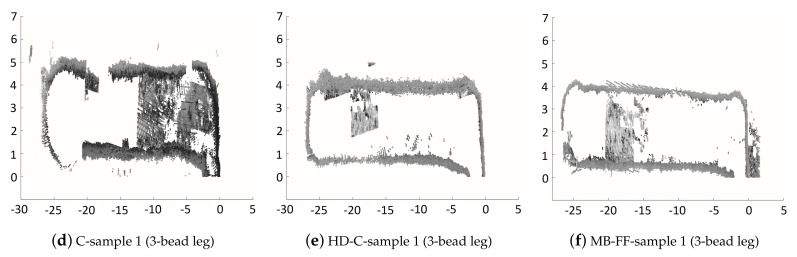
Image of leg section in XY-plane: scatter plot of the XY-plane projection of the point cloud data from the portion of L-shaped structure within height of 21 mm–25 mm. Points A, B, C, and D denote the center location along the length of the 1-bead leg, and the length of the line segment AC and the length of the line segment BD are used to compute the range of leg-width variation. (**a**) C-sample 1 (1-bead leg). (**b**) HD-C-sample 1 (1-bead leg). (**c**) MB-FF-sample 1 (1-bead leg). (**d**) C-sample 1 (3-bead leg). (**e**) HD-C-sample 1 (3-bead leg). (**f**) MB-FF-sample 1 (3-bead leg).

The leg width of each sample has also been examined to understand how each applied laser power strategy affects the hatch width. Figure 11 shows the leg section (in the X–Y plane) images of one representative sample under each laser power strategy. Each X–Y leg section image was generated by (1) slicing the imaging data to obtain the L-shaped segment with height from 21 mm to 25 mm (top portion of the L-shaped structure), and (2) projecting the point cloud data of the L-shaped segment from step (1) at the XY-plane. As a result, the thick boundary (e.g., the line segment $\bar{AB}$ or $\bar{CD}$ in Figure 11) reflects the increase of the leg width when the L-shaped build grows from the height of 21 mm to the height of 25 mm. Table 4 summarizes the corresponding measured range of leg-width at the center location along the leg-length of the L-shaped segments, e.g., for C-sample 1, measured range of leg-width for the 1-bead leg is represented by the length of $\bar{AC}$ and the length of $\bar{BD}$, with the leg width ranging within (1.71, 2.68) mm. Table 4 shows that for the 1-bead leg, the leg width (i.e., hatch width) under *MB-FF* is about 10.5–18.7% less than the hatch width under *C* or *HD-C*; for the 3-bead leg, the leg width under *MB-FF* is about 12.7% less than the leg width under *C* but is comparable to the leg width under *HD-C*.

## 7. Discussions

In this paper, the modeling approximation will inevitably cause errors in estimating the build height, for which Section 6.2 provided model validation and summarized the model prediction errors, and thus subsequently degrades the performance of the resulting model-based controller. Furthermore, the controller in Equation (16) was designed for a nominal case where no modeling uncertainty was considered or quantified. As a result, robustness of the control design is an important issue and needs to be accounted for in the future work. One option is to experimentally evaluate a combination of the nonlinear inverse-dynamics control and a measurement feedback PID control.

The lumped parameter model of the melt-pool dynamics was used in this study for both controller design and closed-loop simulation to generate the simulated closed-loop control trajectory for laser power, which was then implemented as a feed-forward control on the LENS system. The reasonable performance of the resulting control in achieving the target build height implies that the model, although rudimentary due to many assumptions and approximations, is reliable for control design to a certain extent. Nevertheless, future work will look into replacing such simplified model with a high-fidelity FE model in the closed-loop simulation (whereas controller design will still use the lumped-parameter model) to improve the fidelity of the numerical verification of the controller before it is implemented on the physical AM system.

Last, as thermal control is not defined as part of the control objective in this study, experimental measurement of the temperature profiles is not conducted in this paper, and investigation of the thermal behavior (e.g., cooling rate and temperature gradients) and the resulting effects on microstructure of the build part under the *MB-FF* control of the laser power is beyond the scope of this work.

## 8. Conclusions

In this paper, we developed a nonlinear inverse-dynamics controller for laser power and then implemented it as a feedforward controller in depositing L-shaped structures of Ti-6AL-4V on the LENS DED system. Experimental results showed that by applying the proposed feedforward controller for laser power, the resulting build had reduced average height error with respect to the target build height than applying a constant laser power through the entire build or applying a hatch-dependent laser power based on experimental trial and error. Results from this study indicate that such model-based feedforward controller could be a viable option for build-height control in the DED process to replace the experimental trial and error, especially when the real-time, measurement feedback control is not available or too difficult to implement. Future work will investigate using a high-fidelity finite-element simulator on the DED process in the closed-loop simulation for numerical verification of the proposed controller before the resulting control trajectory is implemented in the physical AM system, and it is also worthwhile to experimentally evaluate a combination of the nonlinear inverse-dynamics control and a measurement-feedback PID control.

## Figures and Tables

**Table 1:** Simulation parameters for the open-loop or closed-loop control.

Parameter	Symbol	Value
Density (kg/m${}^{3}$)	ρ	4430
Melting temperature (K)	$T_{m}$	1923
Ambient temperature (K)	$T_{0}$	292
Molten material specific heat (J/kg K)	$c_{l}$	700
Thermal conductivity constant (W/m K)	*k*	6.7
Thermal diffusivity (m${}^{2}$/s)	*a*	$2.4794\times 10^{-6}$
Laser transmission efficiency	η	0.4
Laser scan speed (mm/s)	*v*	10.58
Powder flow rate (kg/s)	*f*	$5\times 10^{-5}$
Hatch spacing (mm)	$s_{h}$	0.8128
Inter-hatch dwell (s)	$t_{h}$	0.097
Inter-layer dwell (s)	$t_{l}$	0.5
Length of each hatch (mm)	*L*	25.4
Melt-pool height-to-width ratio	1/r	0.13
Critical laser power (W)	$Q^{c}$	111.72
Coefficient	β	0.3026
Target build height (mm)	$H_{r}$	25.4
Number of layers	*N*	143
Proportional control gain	$K_{p}$	5000

**Table 2:** Simulated average height (and standard deviation) of each leg of the L-shaped structure under constant 350 W, constant 450 W, or a hatch-dependent 450 W/350 W laser-power strategy.

Laser-Power Applied	Height of 1-Bead Leg	Height of 3-Bead Leg
(1-Bead-Leg/3-Bead-Leg)	(mm)	(mm)
350 W/350 W	22.2 ± 1.3	24.4 ± 1.3
450 W/450 W	28.3 ± 2.0	32.7 ± 1.7
450 W/350 W	26.9 ± 1.4	24.9 ± 1.4

**Table 3:** Measured leg height (average and standard deviation), average error and root mean square error (RMSE) of build height with respect to the target value of 25.4 mm for L-shaped sample structures.

Laser-Power Strategy		*C*		*HD-C*		*MB-FF*	
**Sample No.**		**s1**	**s2**	**s1**	**s2**	**s1**	**s2**
1-bead leg	height (mm)	25.72	25.86	25.96	26.13	25.68	25.90
		(±0.26)	(±0.26)	(±0.27)	(±0.23)	(±0.15)	(±0.13)
	*e*(%)	1.26	1.83	2.19	2.86	1.08	1.95
	RMSE (mm)	0.4109	0.5308	0.6126	0.7606	0.3115	0.5130
3-bead leg	height (mm)	27.76	27.79	27.07	27.35	26.57	26.99
		(±0.55)	(±0.47)	(±0.37)	(±0.40)	(±0.33)	(±0.31)
	*e*(%)	9.27	9.42	6.57	7.67	4.59	6.25
	RMSE (mm)	2.4184	2.4388	1.7081	1.9871	1.2129	1.6185

**Table 4:** Range of measured leg width at the center location along the leg length of the L-shaped segments from height of 21 mm to 25mm; as illustrated in Figure 11a, the range of leg width for the 1-bead leg of C-sample 1 is represented by (dist(A,C), dist(B,D)), where dist(·,·) represents the distance of two points.

Laser Power	Sample No.	Range of Width of 1-Bead Leg	Range of Width of 3-Bead Leg
450 W/450 W	C sample 1	(1.71, 2.68) (mm)	(2.94, 4.09) (mm)
450 W/350 W	HD-C sample 1	(1.73, 2.62) (mm)	(2.65, 3.60) (mm)
MB-FF	MB-FF sample 1	(1.53, 2.18) (mm)	(2.96, 3.57) (mm)

## Data Availability

The data presented in this study are available on request from the corresponding author.
